# Case report: Peptide receptor radioligand therapy in metastatic pediatric neuroendocrine tumors

**DOI:** 10.3389/fnume.2023.1193880

**Published:** 2023-08-03

**Authors:** Khanyisile Hlongwa, Olumayowa Kolade, Abdulilah Alnabulsi, Rachelle Steyn, Anita Brink, Vikas Prasad, Stuart More

**Affiliations:** ^1^ Department of Nuclear Medicine, Red Cross Children’s Hospital and Groote Schuur Hospital, University of Cape Town, Cape Town, South Africa; ^2^ Clinical Theranostics, Department of Nuclear Medicine, Mallinckrodt Institute of Radiology, Washington University, St Louis, MO, United States

**Keywords:** PRRT, neuroendocrine tumor, pediatrics, DOTA-TATE, theranostics (combined therapeutic and diagnostic technology)

## Abstract

Neuroendocrine tumors (NETs) are not commonly diagnosed in children. Metastatic NETs tend to have poor outcomes, and this is seen in adult and pediatric populations. The role of somatostatin receptor imaging using [^68^Ga]Ga-DOTA-TATE for imaging and peptide receptor radionuclide therapy (PRRT) with [^177^Lu]Lu-DOTA-TATE in children is currently not well established. The guidelines for treating pediatric neuroendocrine tumors are still lacking. Extensive trials have been conducted in adult patients and have demonstrated improved survival in metastatic NETs with PRRT using [^177^Lu]Lu-DOTA-TATE. We present two pediatric patients with metastatic NETs who were imaged with [^68^Ga]Ga-DOTA-TATE PET/CT and treated with [^177^Lu]Lu-DOTA-TATE therapy.

## 1. Introduction

Neuroendocrine tumors (NETs) in the pediatric population are not common in occurrence; however, their incidence and prevalence have increased over the last few decades (1). The incidence of NETs in children has been documented as 2.8 per 1 million children according to the SEER registries of 1976–2006, which looked at the incidence, survival, and prevalence of neuroendocrine tumors in children (2). NETs are commonly seen in the midgut in the pediatric population (1, 2). Several treatment options are available according to different NET guidelines; however, none of these are directly aimed at pediatric populations (3–6). The majority of NETs express somatostatin receptors (SSTR), which are targeted by somatostatin analogs (SSA). Novel radiopeptides labeled with SSA are now the method of choice to fully stage and localize the extent of disease in patients with NETs via [^68^Ga]Ga-DOTA-TATE positron emission tomography/computed tomography (PET/CT). The same SSA analog can be coupled in the form of DOTA with [^177^Lu]Lu-DOTA-TATE to provide peptide receptor radionuclide therapy (PRRT) in patients with metastatic well-differentiated NETs (3, 6, 7).

In metastatic non-resectable disease, NETs are managed sequentially with SSA, novel targeted drugs (Everolimus/Sunitinib), chemotherapy, and PRRT (6). Radioligand therapy in pediatrics has been demonstrated with [^90^Y]Y-DOTA-TOC and [^177^Lu]Lu-DOTA-TATE in solid tumors, including NETs; however, no prospective trials have been carried out and evidence is limited (8–10). In this communication, we present and report our experience in treating two pediatric patients with extra-appendiceal gastroenteropancreatic (GEP) NETs. The two patients are both approximately the same age, with high tumor burden, hepatic metastasis, and molecular imaging and therapy with SSTR ligands.

## 2. Patient A

### 2.1. Clinical history and investigations (before PRRT)

A 9-year-old boy presented to the hospital in December 2017 after fainting at school preceded by shortness of breath on minimal exertion a few weeks prior. At presentation, he was noted to be severely anemic, with a hemoglobin of 4.0 g/dL, and in heart failure. He was resuscitated with transfusions. Further evaluation revealed severe iron deficiency and the patient was started on iron supplementation. At the time, chronic infections, gastrointestinal bleeding, and parasite infestation were excluded and the cause was considered to be dietary.

Six months later, there was a second fainting spell, now with a hemoglobin of 3 g/dL at presentation, and more frequent relapses of approximately four more episodes over the next year, each requiring blood transfusion. Bone marrow biopsy showed hyperplastic erythropoiesis secondary to anemia. The iron deficiency was also not corrected despite therapeutic iron supplementation.

An upper gastrointestinal tract endoscopy that was subsequently performed to exclude peptic ulceration revealed a macroscopic gastric ulcer, which was biopsied. The histology showed numerous *Helicobacter pylori* and florid active chronic *Helicobacter pylori* gastritis. A repeat scope 2 months later showed no interval decrease in the lesion macroscopically, despite proton pump inhibitors and *H. pylori* eradication therapy, and numerous *H. pylori* on histology. Zollinger-Ellison syndrome was excluded based on normal serum gastrin levels. Biopsies from endoscopy showed neuroendocrine cells, on account of which a partial gastrectomy was carried out, involving the greater curvature of the stomach and the primary tumor site. Histology demonstrated a transmural infiltrating tumor with a nested growth pattern, with lymphovascular as well as perigastric soft-tissue invasion. There was no discohesive or tubular morphology. A grade 2 neuroendocrine tumor with regional lymph node involvement was diagnosed with the following characteristics: mitotic count=2 per 10 high-power fields; immunohistochemistry=CK7 – patchy, chromogranin A-positive, HEPAR 1-positive, synaptophysin-positive, and Ki-67 5%–8%; staging=pT4a, N1b; and Mx= Stage IIIb.

The patient had no family history of malignancies and genetic screening was not acquired for this patient.

**Figure 1:**
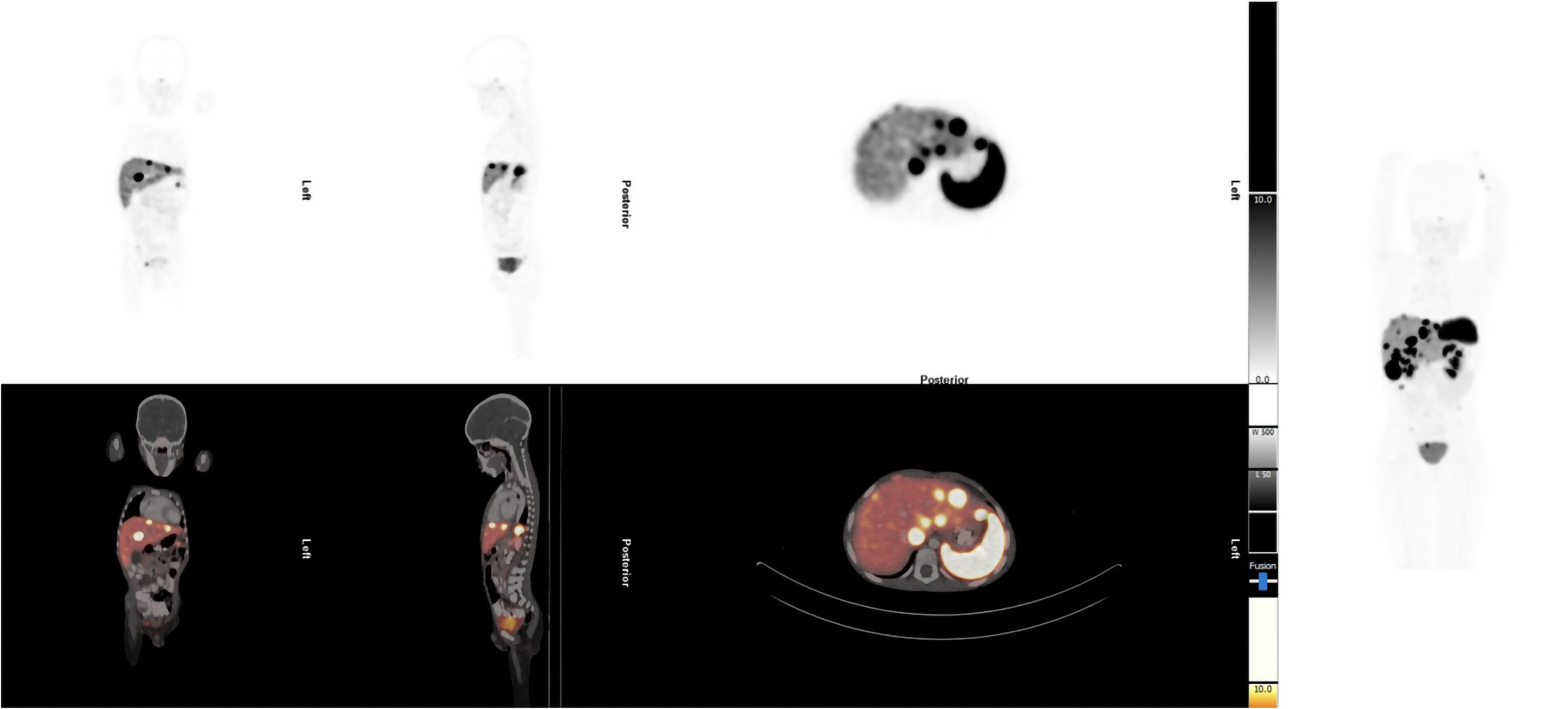
Staging [^68^Ga]Ga-DOTA-NOC PET/CT demonstrated multiple [^68^Ga]Ga-DOTA-NOC avid liver and intra-abdominal lymph node metastases.

The patient had multiple investigations. Abdominal ultrasound showed multiple hypoechoic lesions in the liver that were also seen on CT. A staging [^68^Ga]Ga-DOTA-NOC PET/CT demonstrated multiple [^68^Ga]Ga-DOTA-NOC avid liver and intra-abdominal lymph node metastases (Figure 1). The initial chromogranin A was 37 850 ng/ml (normal: <109); urine 5-hydroxyindoleacetic acid (HIAA): creatinine ratio=10.2 (normal: 0–8.3). Hematological, renal, and liver functions were within normal limits for therapy. Due to the non-availability of somatostatin analogs at his referring hospital, the patient was referred for PRRT with [^177^Lu]Lu-DOTA-TATE therapy.

**Figure 2:**
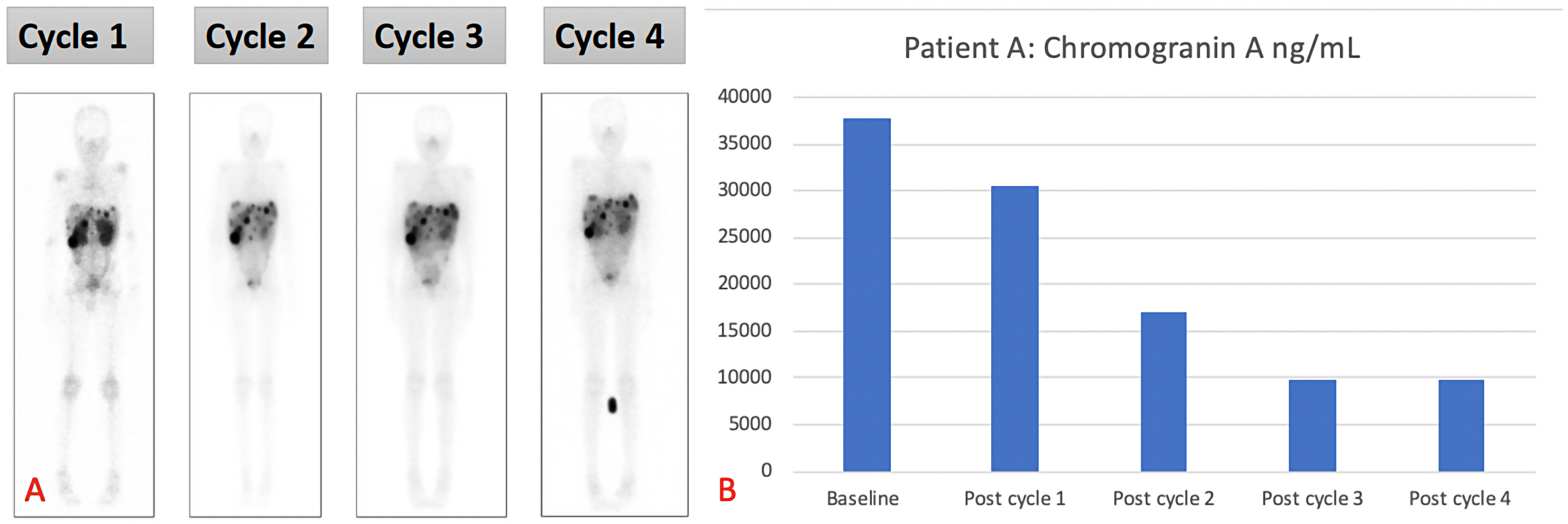
(**A**) Demonstrating post-therapy uptake in whole-body imaging and (**B**) post-therapy tumor markers for patient A.

The patient received four cycles of PRRT (2.5 Giga-becquerels per cycle) (100 MBq/kg) (11), with [^177^Lu]Lu-DOTA-TATE, and had no adverse effects after therapy. The treatment resulted in a biochemical response and stable disease. The patient is still well to date, is growing well, and has not experienced any relapses. The overall survival since the start of therapy is 37 months (Figure 2A demonstrating post-therapy uptake in whole body imaging after each cycle) (12). The uptake in whole-body imaging and Figure 2B show post-therapy tumor markers. The patient’s follow-up radiological imaging demonstrated stable disease. RECIST 1.1 (12) The biochemistry following each cycle has been included in Table 1.

**Table 1:** Biochemistry on follow-up for patient A.

Date	Baseline	Post cycle 1	Post cycle 2	Post cycle 3	Post cycle 4	1-year post Rx	2-year post Rx
Hb	7.3	12.7	23.8	12.4	11.6	12.8	11.6
PLT	637	444	431	274	464	405	396
WCC	847	8.38	9.05	11.78	7.04	7.94	8.02
Urea	2.5	3.1	3.4	2.5	2.8	2.3	3.9
Creatinine	27	25	36	22	24	35	29
Total bilirubin	4	2	3	11	2	–	–
Albumin	46	44	43	37	43	–	–
CGA	37,850	30,560	17,040	9,700	9,716	7,555	4,679

Hb, hemoglobin; PLT, platelets; WCC, white cell count; CGA, chromogranin A.

## 3. Patient B

### 3.1. Clinical history and investigations (before PRRT)

A 9-year-old boy presented with a 5-month history of intermittent chronic abdominal pain, 1 month of intermittent vomiting with occasional hemoptysis, and associated weight loss. He had no other significant medical history except for preterm birth at 34 weeks of gestation. His surgical history included a right inguinal herniotomy in March 2019. On clinical examination, there was hepatomegaly 3 cm below the costal margin and non-significant lymphadenopathy. A liver biopsy confirmed the diagnosis of NET. Histology confirmed a metastatic NET, the primary neoplasm could not be demonstrated on the liver biopsy, the comment being that primary neoplasm in these cases may be inconspicuous or microscopic. The tumor demonstrated a Ki67 of 15% and a mitotic count of 6 per 10 high-power fields. The immunohistochemistry demonstrated the following positive markers: synaptophysin, chromogranin A, CAM5.2, S100, and CD99. The following markers were negative: gastrin, glucagon, insulin, CDX2, HEPAR1, TTF1, and Glypican3.. The patient had no family history of malignancies, and genetic screening was not acquired for this patient.

**Figure 3:**
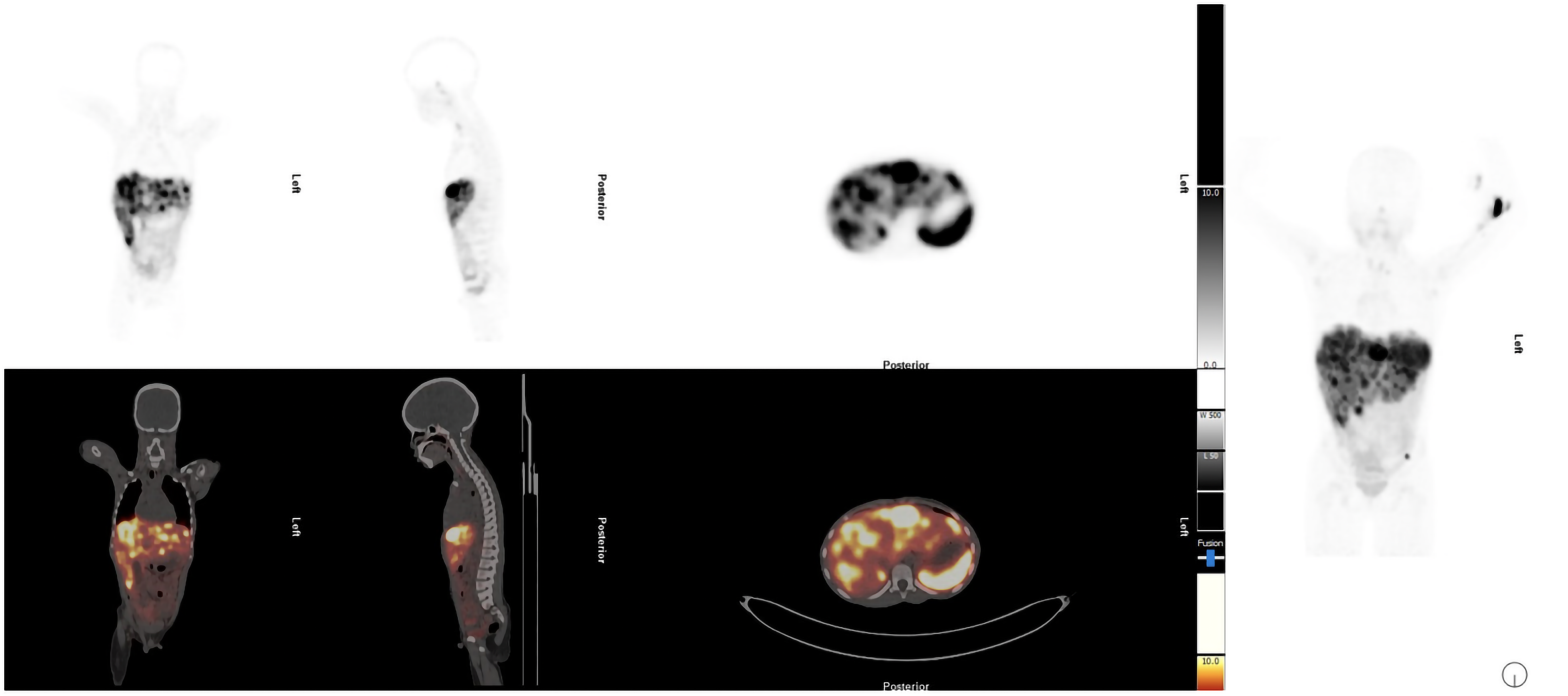
Staging [^68^Ga]Ga-DOTATATE PET/CT demonstrated multiple [^68^Ga]Ga-DOTA-TATE avid liver lesions with no other sites of abnormal uptake.

Abdominal ultrasound showed one hypoechoic lesion of 8 mm × 6 mm in both lobes of the liver with no other abnormal finding. A CT scan of the abdomen demonstrated hepatomegaly with multiple heterogeneous round lesions scattered in the right and left lobes. An underlying non-benign process was suggested for consideration, including metastatic deposits, lymphoma, or multifocal hepatoblastoma. The CT scan of the chest was normal. The liver biopsy demonstrated features in keeping with metastatic NET, Ki-67 of approximately 15%, and a mitotic count of 6 per 10 high-power fields. The baseline chromogranin A was 2,748 ng/mL. Hematological, renal, and liver functions were within the normal limits for therapy. A staging [^68^Ga]Ga-DOTATATE PET/CT demonstrated multiple [^68^Ga]Ga-DOTA-TATE avid liver lesions with no other sites of abnormal uptake (Figure 3). Due to the non-availability of somatostatin analogs at his referring hospital, the patient was referred for PRRT with [^177^Lu]Lu-DOTA-TATE therapy.

**Figure 4:**
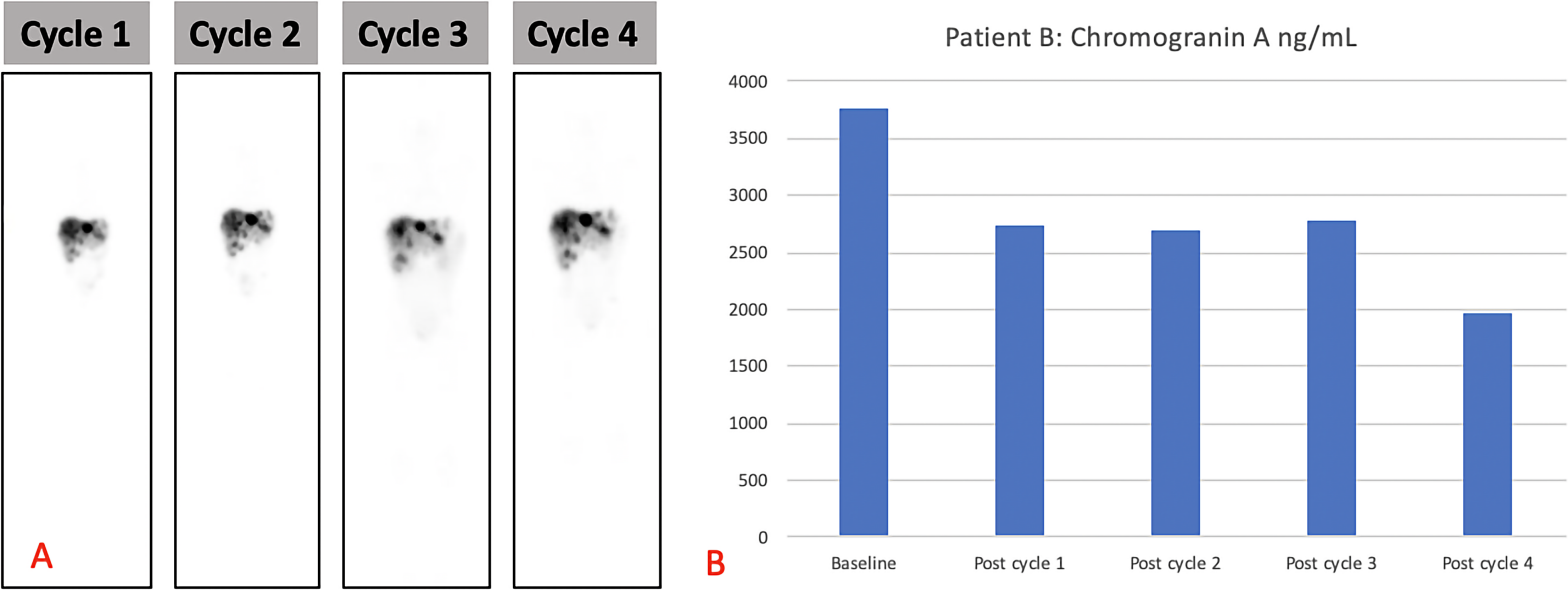
(**A**) Demonstrating post-therapy uptake in whole-body images and (**B**) post-therapy tumor markers for patient B.

The patient received four cycles of PRRT (2.5 GBq/cycle) with [^177^Lu]Lu-DOTA-TATE and had no adverse effects after therapy. The treatment resulted in a biochemical response (Figure 4A shows the post-therapy uptake in whole-body images and Figure 4B shows the post-therapy tumor markers). The patient relapsed 6 months after completion of the four cycles of PRRT and was treated with somatostatin analogs. Somatostatin analogs were available in the state hospital sector by the time of relapse but were not initially available on presentation. He remains stable, is growing well, and is alive to date with an overall survival of 21 months after the initial therapy. His follow-up radiological imaging demonstrated stable disease (RECIST 1.1) (12). The biochemistry results after each treatment cycle are shown in Table 2.

**Table 2:** Biochemistry on follow-up for patient B.

Date	Baseline	Post cycle 1	Post cycle 2	Post cycle 3	Post cycle 4	1-year post Rx
Hb	10.9	9.7	10.1	11.1	10.7	11.0
PLT	455	348	355	337	270	227
WCC	3.62	2.63	5.32	3.48	2.11	2.90
Urea	3.7	3.0	3.1	4.0	4.2	3.8
Creatinine	49	37	30	37	34	36
Total bilirubin	<4	<3	<3	4	4	3
Albumin	42	41	41	46	46	47
CGA	3,769	2,733	2,701	2,780	1,975	2,349

Hb, hemoglobin; PLT, platelets; WCC, white cell count; CGA, chromogranin A.

## 4. Discussion

We report our initial experience with [^68^Ga]Ga-DOTA-TATE/[^177^Lu]Lu-DOTA-TATE theragnostic combination in two children with metastatic NETs. In this report, we have demonstrated a good biochemical response to [^177^Lu]Lu-DOTA-TATE with evidence of stable disease on post-therapy imaging. Our findings demonstrate the feasibility of utilizing [^177^Lu]Lu-DOTA-TATE in pediatric patients with metastatic NET and achieving a durable treatment response. These results are concordant with the case report by Foster et al., who treated two patients of a similar age and demonstrated minimal side effects after therapy, a biochemical response, and stable disease after therapy for one patient (13).

We also demonstrate the utility of the [^68^Ga]Ga-DOTA-TATE/[^177^Lu]Lu-DOTA-TATE theragnostic pair in NETs. The pair has also been used in pediatric patients with refractory metastatic neuroblastoma. The therapy was found to be feasible and well tolerated with limited toxicity (14). Pretherapy imaging was beneficial in that it demonstrated additional disease that was not visualized on standard radiological imaging (14).

Our report underscores that the two children had unique and rather rare presentations for pediatric NETs. Presenting symptoms in pediatric NETs more commonly include features of acute appendicitis, non-specific abdominal pain, and, less commonly, features of carcinoid syndrome (1). No report was found in the literature with a presentation of syncope and anemia as presented in patient A. There is also a low incidence of hepatic metastasis on pediatric NETs demonstrated in the literature, and a high tumor burden at presentation is not common. The more common presentation is midgut NET, even though patients are diagnosed late and often with metastatic disease (1). Both patients A and B, unlike the predominant pattern, had hepatic metastasis and high tumor burden findings that were clearly demonstrated on [^68^Ga]Ga-DOTA-TATE imaging. This important imaging modality also serves as the bedrock for planning PRRT for NETs and has been clearly demonstrated to have significant benefits. PRRT has been demonstrated as a safe procedure with low toxicity to the marrow and kidneys (15). Similar to diagnostic imaging with SSTR, there is also a paucity of literature on PRRT in the pediatric population.

Clinical trials in adults with midgut NETs preliminarily demonstrated improved progression-free survival and evidence of overall survival when using [^177^Lu]Lu-DOTA-TATE (16). However, the final analysis did not reach statistical significance regarding overall survival despite remaining clinically relevant with minimal side effects and toxicity in the long-term follow-up (17). There are various other phase-III clinical trials that aim to establish the role of PRRT in low- and high-grade NETS, namely, the COMPETE trial, which evaluates the safety of [^177^Lu]Lu-Edotreotide in comparison with Everolimus in patients with G1 and G2 pancreatic NETs and GE NETs. Another trial using a similar radionuclide is COMPOSE, which compares [^177^Lu]Lu-Edotreotide as the first- or second-line treatment with the best standard of care in patients with well-differentiated G2 and G3 GEPNETs. Lastly, NETTER-2 compares [^177^Lu]Lu-DOTA-TATE as first-line therapy compared to Octerotide in patients with well-differential G2 or G3 GEP-NETs (18, 19). Prospective trials in pediatric patients with metastatic NET are yet to be established, but the advantage of therapy has been demonstrated in solid tumors, neuroblastoma, and in limited NET (8–10). The limitation of this case series is the absence of dosimetry and the standardization of therapy in pediatric populations. Nevertheless, these cases highlight the benefit of using PRRT in children with metastatic NETs and its potential advantages. Large-scale prospective data will be helpful in order to establish the use of PRRT in the pediatric population.

## Conclusion

The findings of this case series demonstrate that PRRT is a safe and effective option in children with metastatic GEP NETs. Clinical trials in pediatrics need to be established to have a standard of care for this patient population.

## Data Availability

The data analyzed in this study are subject to the following licenses/restrictions: The data presented in this article are not readily available as they include information pertaining to minors. Requests to access the datasets should be directed to Red Cross Children's Hospital/Groote Schuur Hospital Department of Nuclear Medicine. Requests to access these datasets should be directed to stuart.more@uct.ac.za.
